# Draft Genome Sequence of *Streptococcus equi* subsp. *zooepidemicus* Strain S31A1, Isolated from Equine Infectious Endometritis

**DOI:** 10.1128/genomeA.00683-13

**Published:** 2013-09-05

**Authors:** Isabelle da Piedade, Bolette Skive, Henrik Christensen, Anders Miki Bojesen

**Affiliations:** Department of Veterinary Disease Biology, Faculty of Health and Medical Sciences, University of Copenhagen, Frederiksberg C, Copenhagen, Denmark

## Abstract

We present the draft genome sequence of *Streptococcus equi* subsp. *zooepidemicus* S31A1, a strain isolated from equine infectious endometritis in Denmark. Comparative analyses of this genome were done with four published reference genomes: *S. zooepidemicus* strains MGCS10565, ATCC 35246, and H70 and *S. equi* subsp. *equi* strain 4047.

## GENOME ANNOUNCEMENT

*Streptococcus equi* subsp. *zooepidemicus* is a beta-hemolytic Gram-positive Lancefield group C bacterium found in a wide range of hosts, including farm animals, dogs, and humans (1). *S. equi* subsp. *zooepidemicus* appears to be part of the normal bacterial microflora of the upper respiratory tract and caudal reproductive tract of horses and is also found in healthy bodily systems of other species, such as chickens, pigs, and donkeys (2, 3). However, *S. equi* subsp. *zooepidemicus* is also an opportunistic pathogen associated with a wide variety of diseases, e.g., pneumonia, septicemia, mastitis, placentitis, and endometritis (4–6). Human infections are rare, but severe complications associated with *S. equi* subsp. *zooepidemicus* infection have been reported (7). *S. equi* subsp. *zooepidemicus* is the most frequently isolated pathogen from the uteruses of mares (8, 9). It is hypothesized that gene loss and gain in *S. equi* subsp. *zooepidemicus* have shaped its evolution and enabled it to conquer this niche (10). In support of this, pulsed-field gel electrophoresis (PFGE) and multilocus sequence typing (MLST) have shown that *S. equi* subsp. *zooepidemicus* isolated from infectious endometritis in mares appears to belong to a genetically distinct group (11).

*S. equi* subsp. *zooepidemicus* strain S31A1 was isolated from the uterus of a mare with endometritis in Denmark. The genome sequencing of *S. equi* subsp. *zooepidemicus* S31A1 was achieved using Illumina HiSeq 2000 with paired-end reads. A total of 7,482,442 reads with a length of 98 nucleotides (nt) were assembled *de novo* with the program CLC Genomics Workbench (v4.7.2) with default parameters, resulting in 92 reads with an average size of 21,495 bp and coverage of 374×. The resulting 92 contigs (all >3,000 bp) were ordered by alignment to the reference genomes of *S. equi* subsp. *zooepidemicus* MGCS10565 (12), ATCC 35246 (13), and H70 (10) and *S. equi* subsp. *equi* 4047 (10) using Mauve Contig Mover (14). The total size of the resulting assembly is 1,959,199 bp. The draft genome was annotated using Prokka toolbox v1.5.2 (http://www.vicbioinformatics.com/software.prokka.shtml). We identified 1,769 predicted protein-coding sequences and 1,784 gene sequences with average lengths of 304 amino acids and 908 nt, respectively. The G+C content of the S31A1 genome is 41.5%, a value similar to that of the reference genomes (~41%).

With respect to virulence-associated traits, the S31A1 genome contains M-like proteins, immunoglobulin G-binding proteins (G and H), immunoglobulin A receptor, capsule synthesis proteins, the virulence factor EsxA, T6 antigen, and several toxins, like cholera toxin secretion protein. Antimicrobial resistance factors, like penicillin binding proteins and bacteriophage-associated proteins (phage protein C, bacteriophage scaffolding protein D), have also been identified. Ongoing work is being carried out to compare the genomes of other *S. equi* subsp. *zooepidemicus* isolates in order to identify the genetic loci that are implicated in their pathogenesis.

### Nucleotide sequence accession number.

This whole-genome shotgun project has been deposited at DDBJ/EMBL/GenBank under the accession no. AUXA00000000. The version described in this paper is the first version.
